# Water dissociation efficiencies control the viability of reverse-bias bipolar membranes for CO_2_ electrolysis

**DOI:** 10.1038/s44286-025-00306-7

**Published:** 2025-11-17

**Authors:** Gerard Prats Vergel, Huan Mu, Nikita Kolobov, Jasper Biemolt, David A. Vermaas, Thomas Burdyny

**Affiliations:** Department of Chemical Engineering, Faculty of Applied Sciences, Delft University of Technology, Delft, The Netherlands

**Keywords:** Electrocatalysis, Chemical engineering

## Abstract

Bipolar membranes operated under reverse-bias (r-BPM) provide the only potential route to use anodes free of platinum group metals in CO_2_ electrolyzers when paired with the oxygen evolution reaction. Under 100% water dissociation efficiency (WDE) conditions, the OH^−^ generated by a r-BPM fully replenishes the OH^−^ consumed by the oxygen evolution reaction, maintaining an alkaline anolyte. However, unwanted co-ion crossover leads to <100% WDEs, gradually causing anolyte acidification and nickel-based anodes to corrode over time. Here we experimentally measured the WDE of r-BPMs in a membrane–electrode assembly configuration as a function of the current density, anolyte concentration and cation identity, finding that the highest measured WDE of 98% is insufficient to maintain an alkaline environment over extended operation. We further highlight through modeling that WDEs >99.8% are required to operate for >10,000 h with reasonable anolyte volumes. Our results show that r-BPMs CO_2_ electrolyzers require additional strategies, such as reverting to platinum group metal anodes or regenerating the anolyte, to operate stably at an industrial scale.

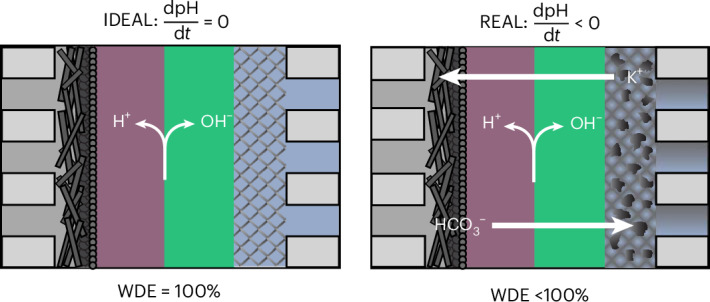

## Abstract (web summary)

Reverse-biased bipolar membranes can enable CO_2_ electrolysis with iridium-free anodes for extended durations, but only if 100% of the ionic charge is carried by water dissociation. Here, the authors show that practical systems fall far below unity water dissociation efficiencies, highlighting a performance gap for sustained alkaline operation using nickel-based anodes.

## Main

Low-temperature CO_2_ electrolysis offers a pathway to replace fossil fuels as raw materials to produce carbon-based products. During the past several years, CO_2_ electrolysis has experienced many advancements that have helped bridge the gap toward its commercialization^1–4^. One of the biggest challenges intrinsic to CO_2_ electrolyzers is the formation of carbonate species from CO_2_ molecules reacting with the locally generated OH^−^. When utilizing anion exchange membranes (AEMs), carbonate negatively impacts the electrolyzer performance through both salt formation^5,6^ and carbonate crossover^7–9^. Crossover results in carbonate accumulation in the anolyte, which drives pH toward neutral conditions. Even when starting with a 1 M KOH solution (pH 14), acidification of the anolyte occurs in just a few hours^10^.

When the anodic reaction is the oxygen evolution reaction (OER), acidification then requires the use of platinum group metals (PGMs) such as iridium because nickel-based anodes (for example, NiFeO_*x*_) will corrode^11,12^. Iridium’s scarcity (annual supply of ~7 tons (refs. ^13,14^)), cost and environmental impact, however, raise concerns about its feasibility for industrial-scale usage. Proton exchange membrane water electrolyzers face a similar iridium dependency, necessitating alternative strategies such as different cell configurations, decreasing catalyst loading, improving the recycling rate of Ir anodes and designing PGM-free catalysts for the acidic anodic OER^15–17^.

**Fig. 1:**
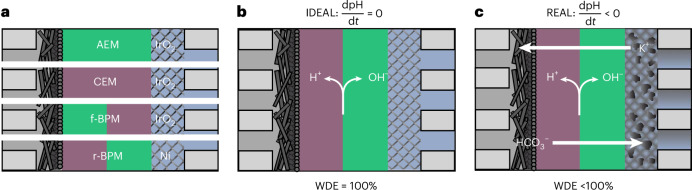
Overview of viable ionic membrane choices for CO_2_ electrolysis and their associated anode material. **a**, The membrane selection defines the anode material. An AEM, CEM or f-BPM will require Ir-based OER catalysts, whereas an r-BPM could use Ni-based anodes. **b**, The reverse-bias BPM under ideal conditions (WDE of 100%) with no co-ion leakage, leading to a constant anolyte pH over time. **c**, The reverse-bias BPM in nonideal conditions (WDE <100%), with carbon and cation crossover causing an anolyte pH decrease that leads to corrosion of the Ni anode.

In a membrane electrode assembly (MEA)-based CO_2_ electrolyzer^18^, the membrane type plays a key role in defining the ion transport inside the cell and subsequently the local electrode environment^19–21^. Here, AEMs currently show the best MEA CO_2_ electrolyzer performance metrics, but as noted, carbonate crossover necessitates iridium for the OER^22^. A cation exchange membrane (CEM) paired with acidic anolyte conditions can inhibit carbon crossover, but owing to the acidic environment, iridium would still be required^23^. Of the four viable membrane configurations shown in Fig. 1a, only a bipolar membrane (BPM) operating in the reversed-bias mode works to maintain an alkaline anolyte environment and thus provides the only potential route for CO_2_ electrolyzers to utilize PGM-free anodes^24^.

A BPM, which consists of an anion exchange layer (AEL) and a cation exchange layer (CEL), can operate in two different modes: forward bias (f-BPM) and reverse bias (r-BPM)^25,26^. Under forward bias, carbonate species from the cathode migrate across an AEL, where they are met with H^+^ migrating across a CEL from an acidic anolyte^27^. These species then convert back to CO_2_ and water, but this requires an acidic anolyte and iridium anode^28–30^. When operated in reverse bias, a BPM dissociates water into H^+^ that goes to the cathode and OH^−^ that goes to the anode^31^ (Fig. 1b). Such a configuration plays two roles: First, the H^+^ transported to the cathode regenerates (bi)carbonates back to CO_2_, increasing the CO_2_ utilization and minimizing the CO_2_ crossover. Second, the hydroxide transported to the anode can be used to replenish the OH^−^ consumed by the OER, thus maintaining an alkaline environment and thus enabling in principle a PGM-free anode^32–35^. Substantial research has focused on the carbon utilization benefits of r-BPMs, while the second advantage of PGM-free anodes has been somewhat taken for granted^36,37^.

To maintain an alkaline environment at the anode, each OH^−^ consumed by the OER must be replenished via the water dissociation reaction (WDR) in the BPM. However, the co-ion crossover of cation and (bi)carbonate species lowers the ionic current from water dissociation, causing an imbalance between OH^−^ generation and consumption. This nonunity water dissociation efficiency (WDE) leads to anolyte acidification over time (Fig. 1c). Previous studies have shown co-ion transport in commercial (Fumatech) BPMs at a rate equivalent to 0.1–4 mA cm^−2^ (refs. ^38–44^), and modeling and experiments have shown that co-ion crossover is related to the limiting current density^45^. Notably co-ion crossover is a common feature for all types of ion-exchange membrane, with membrane thickness and swelling factors resulting in ion selectivity and conductivity trade-offs. Recent developments in bipolar membranes have trended toward more conductive membrane layers and the use of water dissociation catalysts to lower voltages^46–48^. These features lower the membrane resistance but cause a higher limiting current density, suggesting higher crossover rates as a result. Most works, however, only examine co-ion crossover and WDE using liquids on either side of the BPM, but not in an MEA configuration where ionic concentration gradients are substantial. As shown in electrodialysis applications, the crossover also depends on the pH, current density, electrolyte concentration and ion type^38,40^.

The nonzero crossover, and resulting nonunity WDE, then raises the question of what level of cation and (bi)carbonate crossover is acceptable to maintain alkaline conditions at the anode of a r-BPM CO_2_ electrolyzer for industrial lifetimes of 10,000-40,000 h (refs. ^49,50^). By extension, one can ask: What are the system-wide penalties associated with continually replenishing KOH or CsOH anolytes as they slowly acidify? Answering these questions will help elucidate the viability of using r-BPMs for CO_2_ electrolysis and, subsequently, the promise of using PGM-free anodes.

Here, we experimentally probe the degree to which a r-BPM can inhibit co-ion crossover and maintain an alkaline anolyte environment. To aid in this analysis, we provide a methodology to experimentally determine the WDE of a r-BPM MEA CO_2_ electrolyzer under different process conditions. Here, the WDE allows us to understand the distribution of the ionic current crossing a membrane and thus predict anolyte composition changes over time. On the basis of the experimental results, we created a model that simulates the anolyte pH over time and, hence, the long-term stability of nickel-based anodes. In addition, we analyze possible mitigation strategies to address co-ion transport and their trade-offs with other performance metrics such as the cell voltage and selectivity. We thereby present criteria for and context regarding the feasibility of using r-BPM to replace PGM anodes in CO_2_ electrolysis applications.

## Results

### Quantifying the co-ion crossover and WDE in r-BPMEA configurations

To determine the anolyte’s pH stability over time in r-BPM MEA electrolyzers (r-BPMEAs), we need to quantify the co-ion crossover and subsequently the WDE of the membrane during operation. The WDE of a r-BPM can be defined as the ratio between the partial current density of OH^−^ or H^+^ generated by the BPM and the total current density applied (equation (1)). In essence, the net ionic current crossing a BPM must be equal to the electrical current, and at 100% WDE, the ionic current fully comes from the water dissociation species (equation (2)). In the absence of a catholyte, the WDE is a direct measure inferred from changes to the anolyte composition as a result of the co-ion crossover (for example, K^+^ and HCO_3_^−^/CO_3_^2−^)^41^. Extracting the WDE experimentally then allows for its use in predicting the long-term stability of PGM-free anodes by contrasting its value with the consumption of OH^−^ via the OER.

1 $$\mathrm{WDE}[ \% ]=\frac{{j}_{{\mathrm{OH}}^{-}}}{{j}_{\mathrm{tot}}}$$

2 $${j}_{{\rm{tot}}}={{j}_{{{\rm{OH}}}^{-}}+j}_{{\rm{co}}\text{-}{\rm{ions}}}$$

**Fig. 2:**
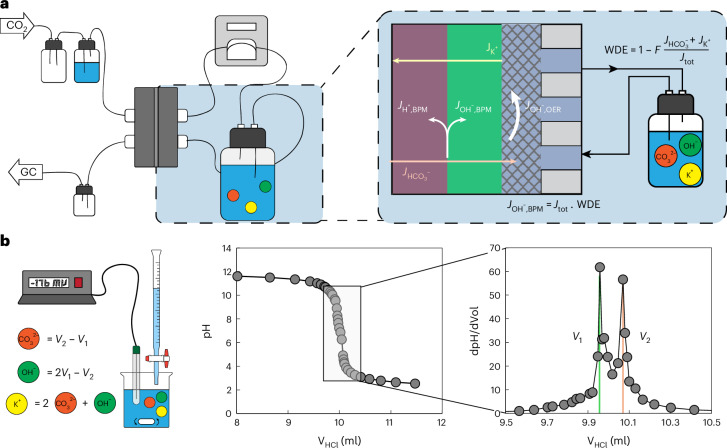
Methodology to estimate the experimental WDE of a r-BPMEA CO_2_ electrolyzer. **a**, CO_2_ electrolysis chronopotentiometry to unmask the ionic transport across the BPM. **b**, Titration to determine the concentrations of (bi)carbonate and hydroxides before and after CO_2_ electrolysis. The cation concentration is determined from electroneutrality. Colored circles represent the ions present in the anolyte. *V*_1_ and *V*_2_ represent the first and second equivalent point of the (bi)carbonate titration, respectively. Source data

To quantify the WDE during CO_2_ electrolysis in an MEA, we performed chronoamperometry experiments and took aliquots of the anolyte to measure the ionic fluxes of potassium and (bi)carbonates (Fig. 2a). Through titration of the anolyte sample (Fig. 2b), we can determine the hydroxide and carbonate concentrations, which gives the average (bi)carbonate flux over a given operating time (Supplementary Note 1). Assuming electroneutrality of the anolyte, the flux of potassium can be determined similarly, allowing for the WDE to be calculated from the co-ion fluxes (equation (3)). Using the WDE, it is then possible to characterize the molar flux of OH^−^ generated by the BPM (${J}_{{\mathrm{OH}}^{-},\mathrm{BPM}}$) (equation (4)).

3 $$\mathrm{WDE}=1-F\,\frac{{J}_{{\mathrm{HCO}}_{3}^{-}}+{J}_{{{\rm{K}}}^{+}}}{{j}_{\mathrm{tot}}}$$

4 $${J}_{{\mathrm{OH}}^{-},\mathrm{BPM}}={J}_{\mathrm{tot}}\times\mathrm{WDE}$$

### The dependence of the WDE on the current, anolyte concentration and cation

**Fig. 3:**
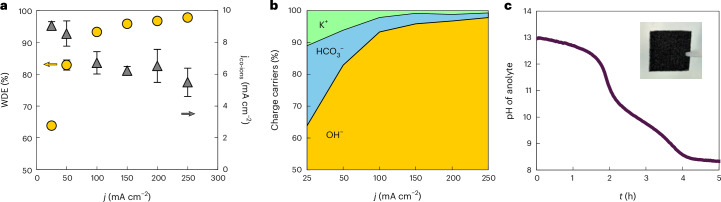
Co-ion leakage causes nonideal r-BPM performance (WDE <100%). **a**,**b**, The effects of the current density (25–250 mA cm^−2^) on the WDE and the partial current density of co-ions, *j*_co-ions_ (**a**) and the charge carrier distribution (**b**). **c**, A short-term CO_2_ electrolysis experiment. The nonideal WDE and the carbonate crossover lead to an anolyte pH drop, which causes corrosion of the Ni anode (and cell failure after only 4 h). The experimental conditions were 20 ml of 0.1 M KOH at 100 mA cm^−2^. Inset: the corroded Ni anode after the experiment. Source data

Using the methodology from ‘Quantifying the co-ion crossover and WDE in r-BPMEA configurations’ section, we quantified the WDE for a commercial Fumasep BPM under current densities from 25 to 250 mA cm^−2^, beginning with a 1 M KOH anolyte (see Supplementary Note 2 for further details). As shown in Fig. 3a, all the current densities exhibited WDE values lower than 100%, with increasing WDE values as the current density was increased. A peak WDE of ~98 % was observed at 250 mA cm^−2^, which is the highest value reported for the different variables analyzed. Such a value implies that, during operation, 98% of the ionic current is provided via the BPM’s WDR while 2% of the ionic flux comes from potassium and (bi)carbonates.

In all the experiments, we observed a co-ion current of cations and bicarbonate ions traveling through the membrane (Fig. 3b) and present a comparative distribution of these ionic fluxes. The respective partial current densities for co-ions fall within the range of 5–10 mA cm^−2^. Note that the crossovers in MEA configurations are slightly higher than those observed in BPMs operating with liquid on either side^40,42,51^. We believe that the higher concentration gradients in MEA cells might lead to the observed crossover rate differences.

**Fig. 4:**
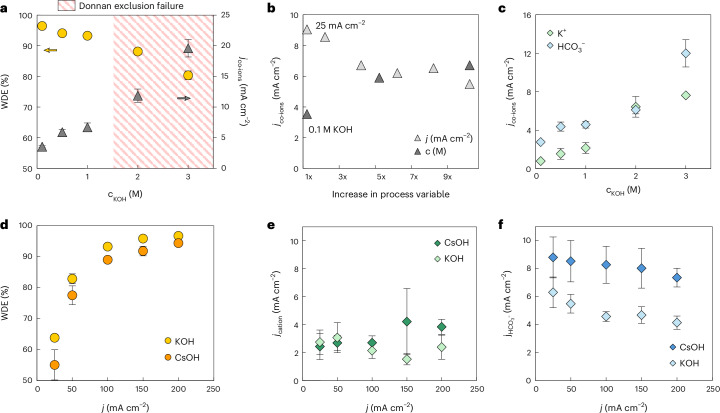
Understanding the co-ion transport in r-BPMEA CO_2_ electrolyzers. **a**, The effect of the KOH concentration on the WDE and *j*_co-ions_. **b**, The sensitivity of *j*_co-ions_ to multiple increases in the current density and anolyte concentration, where ‘1x’ represents 25 mA cm^−2^ or 0.1 M KOH. **c**, The partial current density of K^+^ and HCO_3_^−^ for different anolyte concentrations. **d**–**f**, Revealing the effect of the cation choice (Cs^+^ or K^+^) on the WDE (**d**), and the partial current density of Cs^+^ and K^+^ (**e**) and HCO_3_^−^ (**f**) at current densities of 25–200 mA cm^−2^. Source data

To probe the implications of a given WDE value for anolyte stability, we performed an extended experiment of 4 h at 100 mA cm^−2^ with a small anolyte volume of 20 ml of 0.1 M KOH (Fig. 3c). These conditions resulted in a measured WDE of 96.5% (Fig. 4a), the highest value measured in this work at 100 mA cm^−2^. Here, we found that the Ni anode corroded in less than 4 h, implying that the combination of a r-BPM with a WDE of 96.5%, anolyte volume of 20 ml of 0.1 M KOH and a 5-cm^2^ electrode was greatly insufficient to maintain a stable anolyte as required for the use of PGM-free anodes.

With these base experiments examined, we sought a clearer understanding of the mechanisms of co-ion transport and its dependence on different process variables. We can then see how the WDE can be maximized, while clarifying the trade-offs between minimizing co-ion crossover and improving CO_2_ electrolysis performance metrics (cell voltage and selectivity).

Transport of co-ions, such as potassium and bicarbonate, can occur via two different mechanisms: diffusion, in which the driving force is a concentration gradient, and migration, which is based on transport created by an electric field. The sum of these two transport mechanisms results in the total flux that is measured experimentally. The effect of each individual mechanism was studied by analyzing separately changes in the current density (Fig. 3a) and the concentration gradient (Fig. 4a).

First, the WDE can be maximized by increasing the current density through membrane voltage-induced effects^32,38,44^. From 25 to 100 mA cm^−2^, there is an approximately one-third decrease in the total partial current density of co-ions (Fig. 3a). At higher current densities, the concentration polarization becomes more pronounced, steepening the ionic concentration gradients within the cell. This reduces the effective driving force for co-ion migration, thereby lowering their net crossover^39,52^. In addition, the intensified outward fluxes of H^+^ and OH^−^ from the BPM can also increase the friction with the inward moving co-ions, decreasing their crossover owing to concentrated ion–ion interactions^53–55^.

Beyond 100 mA cm^−2^, the co-ion flux decrease is minimal. Similar behavior has been reported in BPM water electrolyzers (BPMWEs), where increasing the current density from 50 to 500 mA cm^−2^ led to a comparable plateau^54^. After a certain point, increases in the current density no longer strongly enhance the selectivity of the BPM, indicating that membrane voltage effects have saturated. In our results, the co-ion flux can be further minimized by decreasing the anolyte concentration, hence decreasing the K^+^ diffusive flux (Fig. 4a). At 0.1 M KOH, we measured our lowest co-ion fluxes, decreasing the plateau to ~4 mA cm^−2^ and giving a WDE of 96.5% at 100 mA cm^−2^. In contrast, an increase in the KOH concentration (≥2 M) resulted in an abrupt co-ion transport enhancement due to Donnan exclusion failure, recording a WDE of 80% and *j*_co-ions_ of 25 mA cm^−2^ at 3 M KOH. When comparing the sensitivity of the co-ion fluxes to increases in current density and concentration (Fig. 4b), we conclude that both diffusion and migration have a similar impact (of the same order of magnitude) on the co-ion transport in a r-BPMEA CO_2_ electrolyzer and therefore that both make a nonnegligible contribution to the crossover of potassium and (bi)carbonate ions across the BPM.

Isolating the total co-ion partial current densities for each of the ions, we observe a correlation between the K^+^ and HCO_3_^−^ fluxes, as a decrease in K^+^ flux is followed by a decrease in the HCO_3_^−^ flux (Fig. 4c). The ratio between the HCO_3_^−^ and K^+^ fluxes is independent of the current density, being constant at around 2 (Supplementary Fig. 1). However, this ratio increases when decreasing the anolyte K^+^ concentration as the K^+^ fluxes are decreased with respect to those of HCO_3_^−^. These trends are further corroborated when using a 2 M KOH anolyte and varying the current density (Supplementary Fig. 2), implying that the K^+^ diffusive fluxes have a determinant effect on the co-ion transport dynamics. Although these trends can be explained and the correlation between co-ion transport has been observed previously^42^, the reasoning behind the ratio values obtained remains unclear to us, and we suspect that it may be related to the cathode microenvironment and its dependence on the Faradaic efficiency towards CO.

To further examine the role of co-ion transport in our system, we changed the cation in the anolyte to Cs^+^ (Fig. 4d), which also has a positive influence on CO_2_ electrolysis. The use of CsOH resulted in a decrease in the overall WDE measured at each current density. The cationic fluxes of Cs^+^ and K^+^ through the BPM are equal in magnitude, which is due to their similar ion transport properties (Fig. 4e). However, the use of Cs^+^ ions causes an increase in the HCO_3_^−^ fluxes (Fig. 4f), which is responsible for the WDE decrease observed in Fig. 4d. Despite the negative effect of Cs^+^ on the WDE, using Cs ions can positively boost the FE(CO) up to 60–70%, in comparison with the values of 30–40% recorded with K^+^ ions^56–59^ (Supplementary Fig. 3).

In summary, the local concentration of the co-ions plays a key role in the co-ion transport dynamics in the BPM, as both the diffusive and migrative fluxes are dependent on it. From a process point of view, higher current densities and lower anolyte concentrations have been shown to have the best effects in terms of increasing the WDE and thus minimizing the co-ion crossover. However, both have a negative impact on the cell voltage (7 V at 250 mA cm^−2^ and 3 V higher than for an AEM CO_2_ electrolyzer; Supplementary Fig. 4) and CO_2_ reduction selectivity (Supplementary Fig. 5). Similarly, the co-ion transport could also be influenced by tuning the chemical and physical properties of the BPM. Among commercially available BPMs, the Fumasep BPM stands out as one of the best in terms of minimizing the crossover and the WDR overpotentials^40^. However, their low operational limit (<100 mA cm^−2^) imposes substantial challenges when used in electrolysis. Higher currents can be obtained by using state-of-the-art BPMs^46–48^^,60–63^. These advanced BPMs aim to decrease the energy consumption, focusing on WD catalysts to minimize the WDR overpotential and individual layer properties such as thinner layers or a higher ion-exchange capacity to enhance the ionic transport. The impact of such strategies, however, involves a well-known trade-off with an increase of the co-ion transport^64,65^. Therefore, we believe that BPMs with WDEs >99% are unlikely to be realistically applied in an industrially relevant scenario given the trade-offs between WDE, selectivity and cell voltage presented here, especially considering the known need to improve the performance of r-BPM CO_2_ electrolyzers in terms of selectivity and cell voltages^24^.

### Assessing the anolyte stability of r-BPM CO_2_ electrolyzers

The experimental results in the previous section indicated that achieving near-unity WDEs is not possible owing to unavoidable co-ion transport and that near-unity WDEs would probably hurt CO_2_ electrolysis performance metrics. In this section, we ask a different question: What combination of WDE values and anolyte volumes would enable PGM-free anodes to be used over industrially relevant operational times of >10,000 h? While higher WDEs enable higher fractions of OH^−^ to be replenished for the OER reaction, higher anolyte volumes can also ensure that elevated pH conditions are maintained, even as OH^−^ is slowly depleted. To answer these questions, we built a model that predicts the change in the anolyte pH over time as a function of the WDE, anolyte volume, electrolyzer area and current density. We can then define an operational stability window of a Ni-based anode as the time required for the pH of the anolyte to drop below 12^22,23,66,67^.

The model developed consists of a time-dependent mass balance for the different species present on the anode side of the r-BPMEA CO_2_ electrolyzer. The control volume considers the experimental fluxes of the species transported across the BPM ((bi)carbonates, OH^−^ and K^+^) and the tank in which the anolyte is being recirculated. Within the model, we consider carbonate equilibrium, the pH changes related to OH^−^ consumption via OER and OH^−^ regeneration from the BPM (Supplementary Note 3).

**Fig. 5:**
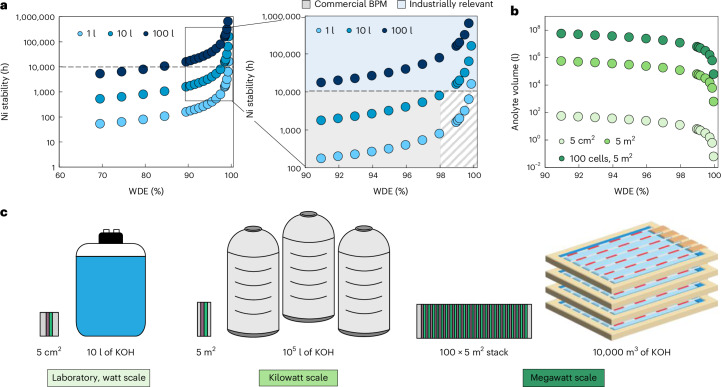
The WDE required for the long-term stability of PGM-free anodes in a r-BPMEA CO_2_ electrolyzer at 200 mA cm^−2^ and 1 M KOH. **a**, The stability of Ni-based anodes (pH >12) for different WDEs and anolyte volumes in a 5-cm^2^ cell. The maximum experimental WDE obtained with a commercial available BPM is ~98%. The diagonal-lined filled area represents WDEs that could not be achieved by commercially available BPMs under the conditions tested. For industrially relevant applications, at least 10,000 h are required. **b**, The volumetric amounts of KOH when scaling up a 5-cm^2^ CO_2_ electrolyzer to 5 m^2^ and a stack 100 × 5 m^2^ for operation for 10,000 h. **c**, An illustration of the magnitude of the volumetric amounts of KOH for a WDE of 98% for laboratory (watt) scale, kilowatt scale and megawatt scale. Source data

As highlighted in Fig. 5a for a 5-cm^2^ cell at 200 mA cm^−2^, the time until Ni anode corrosion varies directly with both the WDE and the utilized anolyte volume. Here, the WDE sets the consumption rate of OH^−^ over a unit of time, while the anolyte volume sets the reservoir of OH^−^ that can be consumed before the pH decreases to the point of Ni corrosion. These results show that, for a 5-cm^2^ cell, a 1 l anolyte volume can reach 10,000-h stabilities only if the WDE >99.8%, which we believe to be an unachievable efficiency, as the highest WDE value measured from our BPM-based MEA was ~98%. Increasing the anolyte volume to 10 l then pushes the minimum WDE threshold to >98% to reach 10,000 h.

These results raise the question of how large the anolyte volume can reasonably be to offset lower WDE values. For this exercise, we also need to consider the total volume of anolyte that would be needed for an industrial-scale electrolyzer, for example, a ~3–5-MW electrolyzer consisting of 100 cells stacked together, each with an active area of 5 m^2^. Figure 5b then compares the volume of anolyte required to operate a r-BPMEA CO_2_ electrolyzer for at least 10,000 h as a function of the WDE for three sizes of electrolyzers. Considering that the maximum WDE obtained experimentally is 98%, a 5-cm^2^ cell then requires 10 l as noted above. While 10 l might not seem like too much when thinking about a 5-cm^2^ laboratory-scale electrolyzer, this translates to 10^7 ^l (10,000 m^3^) for the 100-cell 5-m^2^ electrolyzer. These volumes translate to a consumption of approximately four Olympic swimming pools of KOH after every 10,000 h of operation (Fig. 5c), equal to 24 m^3^ day^−1^ for a single 3–5-MW electrolyzer. These quantities represent both a substantial volume and consumption rate of KOH that appear prohibitive without much higher WDE values or on-site anolyte regeneration.

One potential route to bring down the volume of anolyte needed to a reasonably sized electrolyte tank is to periodically regenerate the anolyte. The regeneration process would again utilize BPMs, but in the form of BPM electrodialysis (BPMED), which uses a pH swing (Supplementary Fig. 6). Briefly, the anolyte is made acidic in the BPMED process, causing the inorganic carbon species to be removed as CO_2_ gas from the electrolyte. Subsequently, the pH is brought back to alkaline state in the BPMED process, which results in a regenerated KOH solution, similar to in BPMED-based direct air capture^68,69^. Anolyte regeneration comes with its own energy and system costs, which would be proportional to the achievable WDE, but it would enable a cyclical process without constant fresh KOH.

Here, we briefly estimate and compare the energy costs of the r-BPMEA CO_2_ electrolyzer versus the BPMED regeneration unit. We assume from literature that a BPMED unit requires between 250 and 1,000 kJ molCO_2_ extracted^70–72^, equating to ~2.3–8.9 MJ kg^−1^ of KOH electrolyte regenerated (where 1 mol CO_2_ released is equivalent to 2 mol KOH regenerated). Meanwhile a r-BPMEA CO_2_ electrolyzer operating at a cell voltage of 3.5 V and an FE(CO) of 60% requires ~40 MJ kgCO produced. Supplementary Fig. 7 shows the ratio of CO produced versus KOH consumed as a function of the WDE for an FE(CO) of 60% (see Supplementary Note 4 for the derivation of the ratio). For the case with a WDE of 90%, 1.1 kg of KOH is required for every 1 kg of CO produced. Using the above energy values, at these conditions, the regeneration unit would then require about 6–25% of the energy used by the CO_2_ electrolyzer. Higher WDE and FE(CO) values would decrease these energy requirements linearly and inversely, respectively.

Combining all of the above analysis together, we see substantial challenges in a r-BPM system being practically used with PGM-free anodes. Co-ion crossover is unlikely to be eliminated to the degree required for KOH consumption to be acceptable from a pure anolyte consumption perspective (>99.8%), and current BPMs currently achieve WDE values <98%. Even higher WDE values are seen to negatively impact the CO_2_ reduction performance. We then conclude that any membrane choice for CO_2_ electrolyzers (AEM, CEM, f-BPM or r-BPM) would necessitate regeneration of the anolyte or the use of acidic and neutral pH-stable anodes such as IrO_2_.

At first glance, we may expect that the issue of anode stability and PGM anodes could be negated by switching away from the OER to an alternate anodic reaction such as glycerol oxidation. However, many aqueous anodic reactions still consume OH^−^ in alkaline conditions, driving the pH to acidic or neutral conditions again^73^. Once in this regime, PGM-free electrodes such as transition metals and carbon are again unstable. Therefore, we expect that the co-ion leakage challenges identified here for r-BPM CO_2_ electrolyzers will also arise when coupled with other anodic reactions.

Reverse-bias BPMs are still a configuration that limits CO_2_ crossover, improves utilization and partially decreases the transport of contaminants such as leached ions from corroded Ir-based or PGM-free anodes. However, these benefits are unlikely to offset the substantial added voltage requirements alone. Reverse-bias BPM CO_2_ electrolyzers incur higher costs than other membrane configurations owing to their greater electricity expenses, relatively low achievable current densities and consequently larger electrolyzer size, which translates into higher material replacement costs^74^. In addition, it is reported that the extra voltage required for the WDR will not justify the related energy expenses to prevent crossover and that an anodic downstream gas separation is most likely to be less energy consuming^75^.

While not the central focus of this work, these volume discussions provide reflection on laboratory-scale experiments that commonly use anolyte volumes on the order of 0.1 l for a 5-cm^2^ electrolyzer. As recently raised in other works, electrolyte tank costs are being overlooked when estimating flow batteries economics^76^. So far, to our knowledge, no work has considered the importance of the dimensions of anolyte tanks in CO_2_ electrolysis. We believe that this is a must to bridge the gap between laboratory research and industrial feasibility. Specifically, in cases such as in r-BPMEA, we show that stability claims are based on the magnitude of the utilized volume, emphasizing the importance of reasonably matching anolyte volumes with electrochemical cell dimensions. This will facilitate the translation of laboratory-scale experiments to more industrial processes and raise awareness of what are realistic experimental conditions for applied research. Additionally, even for systems such as AEM CO_2_ electrolyzers, the overuse of electrolyte volumes may be masking myriad stability or scale-up issues that are then only observed in pilot plants.

## Discussion

This work addresses the general assumption in literature that reverse-bias BPM can enable the use of PGM-free anodes in CO_2_ electrolyzers. We have shown that the WDE can be used to characterize the black box of a r-BPMEA CO_2_ electrolyzer and predict the long-term stability of anolytes that are required for PGM-free anodes. The WDE makes a nonnegligible contribution to the process design for a r-BPM CO_2_ electrolyzer, as its value is directly linked to the amount of anolyte required as a reactant for the anodic OER. WDE values 99.8% are required to avoid additional measures in anolyte treatment. However, we have reported a maximum WDE value of 98% for commercially available BPMs. We believe that eliminating crossover to the extent required is unrealistic, as a trade-off with other cell performance metrics, such as cell voltage and CO_2_R selectivity, is found. Consequently, we conclude that, independently of any membrane type chosen, CO_2_ electrolyzers at an industrial scale will require additional anolyte treatments or operate at neutral or acidic conditions, requiring the use of catalysts that are stable under these conditions.

## Methods

### CO_2_ electrolysis

CO_2_ electrolysis experiments were performed in a 5-cm^2^ MEA cell from Dioxide Materials. The cell was assembled with a cathode, an anode and a membrane, compressed with a torque of 2.5 N m. The cathode was prepared by sputtering 100 nm of Ag onto a Sigracet 39BB gas diffusion layer. For the anode, nickel foam (Ni-4753.005 from Recemat BV) was used. A bipolar membrane (Fumasep FBM, Quintech) in reverse bias separated the cathode and the anode. The BPM employed, with a thickness of ~130–160 µm and a maximum operating current density of 100 mA cm^−2^, consisted of a CEL of sulfonated crosslinked poly-ether ether ketone and an AEL made of polysulfone with quaternary ammonium groups.

The electrochemical setup was connected to our electrolysis standard setup equipped with a potentiostat (PARSTAT MC), mass flow meter and controller and gas chromatograph (GAS compact 4.0)^18^. Humidified CO_2_ was supplied to the inlet at the cathode side at a constant flow rate of 50 s.c.c.m. The cathodic outlet volumetric flow was quantified by a mass flow meter, and its composition was analyzed by the gas chromatograph to determine the CO_2_ electrolysis Faradaic efficiencies (Supplementary Note 5). Similarly, at the anode side, an alkaline electrolyte (KOH or CsOH) was continuously flowed and recirculated at 20 ml min^−1^.

### Determination of the WDE

The WDE was experimentally determined under different process conditions involving a range of current densities, concentrations and anolyte identity (Supplementary Note 3). To guarantee good reproducibility within all experiments, WDE determinations were performed at a constant current density for 1 h with an anolyte volume of 90 ml.

The WDE was obtained from the concentration of the cation (K^+^ or Cs^+^), the carbon species (CO_3_^2−^) and the hydroxide (OH^−^) in the anolyte before and after performing CO_2_ electrolysis. From the difference in concentration between the samples (before, ${c}_{i,\mathrm{co}\text{-}\mathrm{ion}}^{0}$ and after, ${c}_{i,\mathrm{co}\text{-}\mathrm{ion}}^{\mathrm{end}}$), the molar fluxes for each of the species ‘*i*’ (*J*_i,co-ion_) are obtained by using equation (5) and then plugged into the WDE equation (equation (3)). *V*_tank_, *A* and *t*_CP_ refer, respectively, to the anolyte volume, the electrochemical active area and the electrolysis operation time.

5 $${J}_{i,\mathrm{co}\text{-}\mathrm{ion}}=\frac{\left({c}_{i,\mathrm{co}\text{-}\mathrm{ion}}^{\mathrm{end}}-{c}_{i,\mathrm{co}\text{-}\mathrm{ion}}^{0}\right)\cdot {V}_{\mathrm{tank}}}{A\cdot {t}_{\mathrm{CP}}}$$

### Determination of the CO_3_^2−^, K^+^ and OH^−^ concentrations in the anolyte

Warder’s acid–base titration^77^ was used to determine the concentration of CO_3_^2−^ and OH^−^ in the anolyte. Since the initial volume was high enough (90 ml) and the pH of the anolyte remains highly alkaline (pH >12), we assumed that all carbon measured is in the form of CO_3_^2−^. The titrations were performed using a 848 Titrino Plus titrator (Metrohm) with a 0.1 N HCl standardized solution (${c}_{\mathrm{HCl}}$) as titrant. Supplementary Fig. 8 shows an example of a before and after electrolysis titration curve. In the zoom-in curves, one can identify the existing equivalence points for each of the cases.

Before CO_2_ electrolysis, we assumed that no carbonate was present (equation (6)) as anolyte was freshly prepared, and that only one equivalent point (${V}_{\mathrm{EP},0}$) was obtained from the neutralization of H^+^ with OH^−^ (equation (7)). ${V}_{\mathrm{titrate}}$ is the volume of anolyte sample taken for titration, ranging from 1 to 10 ml depending on the original anolyte concentration.

6 $${c}_{{\mathrm{CO}}_{3}^{2-}}^{0}=0$$

7 $${c}_{{\mathrm{OH}}^{-}}^{0}=\frac{{V}_{\mathrm{EP},0}{\rm{\cdot }}{c}_{\mathrm{HCl}}}{{V}_{\mathrm{titrate}}}$$

On the other hand, after CO_2_ electrolysis, the concentration of both CO_3_^2−^ ${c}_{{\mathrm{CO}}_{3}^{2-}}^{\mathrm{end}}$ and OH^−^ ${c}_{{\mathrm{OH}}^{-}}^{\mathrm{end}}$ (equation (8) and (9), respectively) can be obtained directly from the volume of titrant used for both equivalence points (${V}_{\mathrm{EP},1}$ and ${V}_{\mathrm{EP},2}$). ${V}_{\mathrm{EP},1}$ corresponds to the equivalence point between CO_3_^2−^ and HCO_3_^−^, whereas ${V}_{\mathrm{EP},2}$ represents the equivalent point of CO_2_ and HCO_3_^−^.

8 $${c}_{{\mathrm{CO}}_{3}^{2-}}^{\mathrm{end}}=\frac{\left({V}_{\mathrm{EP},2}-{V}_{\mathrm{EP},1}\right){\rm{\cdot }}{c}_{\mathrm{HCl}}}{{V}_{\mathrm{titrate}}}$$

9 $${c}_{{\mathrm{OH}}^{-}}^{\mathrm{end}}=\frac{\left({2{\rm{\cdot }}V}_{\mathrm{EP},1}-{V}_{\mathrm{EP},2}\right){\rm{\cdot }}{c}_{\mathrm{HCl}}}{{V}_{\mathrm{titrate}}}$$

The K^+^ concentrations ${c}_{{\mathrm{K}}^{\mathrm{+}}}$ can then be obtained by applying the principle of electroneutrality (equation (10)).

10 $${{c}_{{{\rm{K}}}^{+}}=c}_{{\mathrm{OH}}^{-}}+2{\rm{\cdot }}{c}_{{\mathrm{CO}}_{3}^{2-}}$$

## Supplementary information

Supplementary Information Supplementary Notes 1–5 and Figs. 1–10.

Supplementary Data 1. Source data for Supplementary Information.

Supplementary Code 1. Python scripts for the model developed.

## Source data

Source Data Fig. 2. Titration curve data.

Source Data Fig. 3. Statistical process data from experiments.

Source Data Fig. 4. Statistical process data from experiments.

Source Data Fig. 5. Simulated data from model.

## Data Availability

The raw and processed data used for this study are available via the 4TU.ResearchData repository at 10.4121/5c4fc325-a71b-4ca8-9c1b-01c0405936a2 (ref. ^78^). Source data are provided with this paper.

## Code availability

The model developed in this work as a Python script is provided in the Supplementary Code.
