# Supplementary material for: Water dissociation efficiencies control the viability of reverse-bias bipolar membranes for CO2 electrolysis
Source: Nat Chem Eng. 2025 Nov 17;2(11):676–84. doi: 10.1038/s44286-025-00306-7 (PMC12638254; doi:10.1038/s44286-025-00306-7)
Supplement: Supplementary file 1 — Supplementary Notes 1–5 and Figs. 1–10. [file 44286_2025_306_MOESM1_ESM.pdf]

nature chemical engineering

Supplementary information https://doi.org/10.1038/s44286-025-00306-7

Water dissociation efficiencies control the viability of reverse-bias bipolar membranes for CO_2 electrolysis

In the format provided by the authors and unedited

Table of Contents

Supplementary Notes 2

Supplementary Note 1 – Derivation of the WDE formula 2

Supplementary Note 2 – WDE process conditions list 4

Supplementary Note 3 - Modelling the anolyte pH evolution over time 4

Supplementary Note 4 - Derivation of the Ratio of CO Produced to KOH Consumed 7

Supplementary Note 5 - CO2 electrolysis performance evaluation 8

Supplementary Data 9

References 14

Supplementary Notes

Supplementary Note 1 – Derivation of the WDE formula

The WDE can be defined as the ratio between the partial current density of OH^- or H^+ generated by the BPM and the total current density applied:

$$WDE\ [\%] = \frac{j_{OH^-,BPM}}{j_{tot}} \tag{1}$$

At any point of the electrolyzer, the total ionic current is equal to the sum of the partial ionic currents for all species present:

$$j_{tot} = \sum_i j_i \tag{2}$$

Assuming that in the AEL the only charged species are OH^- and co-ions (cation from anolyte and carbon species), $j_{OH^-,BPM}$ can be obtained :

$$j_{OH^-,BPM} = j_{tot} - j_{co-ions} \tag{3}$$

The WDE equation can be translated from partial current densities to molar fluxes by using the Faraday's law of electrolysis:

$$J_i = \frac{j_i}{|z_i|F} \tag{4}$$

If species *"i"* is charged, the total flux can be decomposed in the diffusive and migrative flux (Eq. 5). Consequently, we assume that the flux measured experimentally corresponds to both diffusion and migration (Eq. 6), and that both mechanisms are fully captured by j_i in the WDE equation (Eq. 1).[1]

$$J_i = \frac{j_i}{z_i F} = \frac{j_{d,i}}{z_i F} + \frac{j_{m,i}}{z_i F} \tag{5}$$

$$j_{tot} = \frac{F^2}{RT} \cdot \frac{\partial \phi}{\partial x} \sum_i z_i^2 D_i C_i + F \sum_i z_i D_i \frac{\partial C_i}{\partial x} \tag{6}$$

Combining Eq. 1, 3 and 4, we can obtain the WDE as a function of the experimentally determined fluxes and current density used:

$$WDE\ [\%] = 1 - F\,\frac{\sum_i z_i J_{i,co-ion}}{j_{tot}} \tag{7}$$

Where J_i refers to the ionic flux of the i co-ion ($mol \cdot m^{-2} \cdot s^{-1}$), j_i to the partial current density of i co-ion ($A \cdot m^{-2}$), F is the Faraday constant (96485 $C \cdot mol^{-1}$), z_i the absolute value of the charge of the i ion, and j_{tot} the applied current density during chronopotentiometry.

For our specific case, Eq. 7 can be transformed to Eq. 8, assuming the only co-ions present crossing the CEL of the BPM are HCO_3^- and K^+. We hypothesize that the carbon specie entering the CEL is HCO_3^-, which can be converted to H_2CO_3 or CO_3^{2-} with the respective fluxes of H+ and OH- in the CEL or AEL respectively. Our hypothesis is based on the modelling results from recent works r-BPMEA CO_2 electrolyzers[2,3], in which they simulated the pH profile close to the interface between the cathodic catalyst layer and the CEL from the BPM. Although, the models include the addition of an ionomer with the catalyst, the CEL surface pH values reported are always below 6. Therefore, we discard the possibility of any CO_3^{2-} existing in the vicinity of the CEL from the BPM due to the acidic conditions (Supplementary Fig. 9a). Consequently, only molecular CO_2 (dissolved CO_2) or bicarbonate (HCO_3^-) can exist and be the carbon species crossing the CEL. Since dissolved CO_2 is uncharged and has low solubility in the hydrophilic ion exchange membranes we hypothesized and assumed that HCO_3^- is solely responsible for carrying carbon from the cathode to the BPM CEL (Supplementary Fig. 9b). Importantly, even though the WDE value is a function of the carbon specie chosen, it does not have any effect on the model developed as H^+ or OH^- consumption are taken into account as part of the carbonate equilibrium (Supplementary Fig. 9c).

$$WDE\ [\%] = 1 - F\,\frac{J_{HCO_3^-} + J_{K^+}}{j_{tot}} \qquad (8)$$

The experimental fluxes can be derived from the concentrations difference before and after CO_2 electrolysis, $c_{i,co-ion}^{0}$ and $c_{i,co-ion}^{end}$ respectively (Eq. 9). The concentrations are obtained from the respective titrations (Eq. 6-10 in manuscript).

$$J_{i,co-ion} = \frac{\left(c_{i,co-ion}^{end} - c_{i,co-ion}^{0}\right) \cdot V_{tank}}{A \cdot t_{CP}} \qquad (9)$$

V_{tank} is the volume of anolyte used, t_{CP} is the duration of the chronopotentiometry test, A the electrochemically active area.

Supplementary Note 2 – WDE process conditions list

We have determined the WDE under different process variables, such as current density, concentration and cation identity. This refers to the data plotted in Fig. 3 and 4 in the manuscript. For each of the variables studied, each WDE is determined at least three times. For the ease of comparison, for each of the blocks all the other variables have been maintained constant as following:

- Current density: base anolyte 1 M KOH;
  - Variations: 25, 50, 100, 150, 200, 250 mA·cm^{-2}
- Concentration: base current density 100 mA·cm^{-2};
  - Variations: 0.1, 0.5, 1, 2, 3 M KOH
- CsOH: base anolyte 1 M CsOH;
  - Variations: 25, 50, 100, 150, 200 mA·cm^{-2}

Supplementary Note 3 - Modelling the anolyte pH evolution over time

The objective of the model is to use the experimental measured WDE to extrapolate long-term operation of a PGM-free r-BPM CO_2 electrolyzer. The model proposed consists of a simple transient species balance of the ionic species evolving in the anolyte tank over time. The species considered include OH^-, CO_3^{2-}, HCO_3^-, CO_2 and H^+. The modelling of cationic species such as K^+ or Cs^+ has been neglected. The anolyte tank has been modelled as a Batch reactor as we assume the anolyte is being recirculated. That means there is no net flow in/out the tank, and only the generation/consumption of species and its accumulation is considered for the species balance (Eq. 10-14). Therefore, the control volume is limited to the anolyte tank and the ionic fluxes across the BPM obtained experimentally. Thus, the cathodic reactions and phenomena are ignored. Since there is no net water consumption in a CO_2 electrolyzer, and any water transport away from the anode would most likely result in the accumulation of water at the cathode (e.g: leading to the flooding of the GDE), the volume of the tank and the density of the anolyte are assumed to remain constant. The concentration (c_i) for each of the *i* species can be obtained by dividing the mole (n_i) obtained from solving the system of ordinary differential equations (Eq. 10-14), with the volume of the tank (V_{tank}).

$$\frac{\partial n_{OH^-}}{\partial t} = -N_{OER} + N_{BPM} - R_3 - R_4 + R_w \qquad (10)$$

$$\frac{\partial n_{CO_3^{2-}}}{\partial t} = R_2 + R_4 \quad (11)$$

$$\frac{\partial n_{HCO_3^-}}{\partial t} = R_1 - R_2 + R_3 - R_4 + N_{HCO_3^-,BPM} \quad (12)$$

$$\frac{\partial n_{CO_2}}{\partial t} = -R_1 - R_3 \quad (13)$$

$$\frac{\partial n_{H^+}}{\partial t} = R_1 + R_2 - R_w \quad (14)$$

In which R_i correspond to the kinetic molar flows of each carbonate reaction (Eq. 23-27), N_{OER} and N_{BPM} to the molar flux ($mol \cdot s^{-1}$) equivalent to the consumption of OH- via OER and generation of OH- from the BPM respectively (Eq. 15-16), and $N_{HCO_3^-}$ to the molar flux of HCO_3^- crossing the BPM (Eq. 17).

The input variables of the model are: current density ($mA \cdot cm^{-2}$) and active area (cm^2), anolyte volume (L), operation time (h), initial KOH concentration (M) and WDE with the respective co-ion fluxes ($J_{HCO_3^-}$ and J_{K^+}). It is assumed that the WDE is constant over time. The ratio HCO_3^-/K^+ is assumed to be independent of current density and equal to 2 (as demonstrated in Supplementary Fig. 1a) for the several simulations run at 1M KOH over different WDE. However, it can be easily adjusted and tuned to each experiment with specific WDE and $J_{HCO_3^-}$ determined experimentally (Eq. 17).

$$N_{OER} = \frac{j_i}{F} \quad (15)$$

$$N_{BPM} = \frac{j_i}{F} \cdot WDE \quad (16)$$

$$N_{HCO_3^-} = \frac{j_i}{F} \cdot (1 - WDE) \cdot \frac{J_{HCO_3^-}}{J_{HCO_3^-} + J_{K^+}} \quad (17)$$

To account for the carbonate equilibrium (Eq. 18-22) the kinetic constants are used to calculate its reaction rates (Eq. 23-27). As shown in Supplementary Fig.10, not considering the carbonate crossover and therefore its buffer capability results in an overestimation of the stability of the system, therefore it is critical to predicting long-term pH changes.

$$CO_2 + H_2O \overset{k_1,k_{-1}}{\longleftrightarrow} H^+ + HCO_3^- \quad (18)$$

$$HCO_3^- \xleftrightarrow{k_2,k_{-2}} H^+ + CO_3^{2-} \quad (19)$$

$$CO_2 + OH^- \xleftrightarrow{k_3,k_{-3}} HCO_3^- \quad (20)$$

$$HCO_3^- + OH^- \xleftrightarrow{k_4,k_{-4}} H_2O + CO_3^{2-} \quad (21)$$

$$H_2O \xleftrightarrow{k_w,k_{-w}} H^+ + OH^- \quad (22)$$

The reaction rates in $mol{\cdot}s^{-1}$ for each of the previous reactions are represented in Eq. 23-27. The kinetic constants have been obtained from [4] and are shown in Table 1.

$$R_1 = \left(k_1 \cdot c_{CO_2,aq} - k_{-1} \cdot c_{H^+} \cdot c_{HCO_3^-}\right) \cdot V_{tank} \quad (23)$$

$$R_2 = (k_2 \cdot c_{HCO_3^-} - k_{-2} \cdot c_{H^+} \cdot c_{CO_3^{2-}}) \cdot V_{tank} \quad 24)$$

$$R_3 = (k_3 \cdot c_{CO_2,aq} \cdot c_{OH^-} - k_{-3} \cdot c_{HCO_3^-}) \cdot V_{tank} \quad (S25)$$

$$R_4 = (k_4 \cdot c_{HCO_3^-} \cdot c_{OH^-} - k_{-4} \cdot c_{CO_3^{2-}}) \cdot V_{tank} \quad (26)$$

$$R_w = (k_w - k_{-w} \cdot c_{H^+} \cdot c_{OH^-}) \cdot V_{tank} \quad (27)$$

Table 1. Buffering carbonate rate constants.[4]

RATE CONSTANT	VALUE	UNIT
$\boldsymbol{k_1}$	$3.71 \cdot 10^{-2}$	s^{-1}
$\boldsymbol{k_2}$	59.44	s^{-1}
$\boldsymbol{k_3}$	$2.23 \cdot 10^{3}$	$M^{-1}s^{-1}$
$\boldsymbol{k_4}$	$6 \cdot 10^{9}$	$M^{-1}s^{-1}$
$\boldsymbol{K_1}$	$10^{-6.37}$	M
$\boldsymbol{K_2}$	$10^{-10.32}$	M

The moles of CO produced by the r-BPMEA CO_2 electrolyzer can be obtained by using the Faraday electrolysis law (Eq. 28), and can be converted to kg by using its molar mass (Eq. 29).

$$n_{CO} = \frac{j_{CO} \cdot A}{z_{CO_2R} \cdot F} \cdot t \quad (28)$$

$$m_{CO} = n_{CO} \cdot M_{w,CO} \quad (29)$$

Where n_{CO} and m_{CO} the moles and kg of CO respectively, j_{tot} the total current density applied ($A{\cdot}cm^{-2}$), A the electroactive area (cm^{-2}), $z_{CO_2R} = 2$ the number of electrons involved in the reaction, F the Faraday constant and t the time of operation of the electrolyzer.

Supplementary Note 4 - Derivation of the Ratio of CO Produced to KOH Consumed

As a means of determining the comparative energy cost of regenerating an alkaline solution versus the electrolytic production of CO, it is useful to relate the production of CO versus the consumption of KOH. This ratio can be derived from the definition of the WDE, and the assumption that bicarbonate is the primary carbon species crossing the membrane (as opposed to carbonate or carbonic acid). Note that this assumption only impacts the coefficients of the below formulas and not the resulting values, which would be changed for a different carbon assumption. We then begin with Eq. 30 below which describes the OH^- lost in the anolyte due to non-unity WDE's and the loss of hydroxide due to bicarbonate to carbonate conversion.

$$j_{OH^-,lost} = j_{tot} - j_{OH^-,BPM} + j_{OH^-,carbonate} \quad (30)$$

Assuming from the experimental results from Supplementary Fig.1a that 2/3 of the unwanted co-ion transport is related to bicarbonate, and 1/3 to cations, we can transform Eq. 30 to Eq. 31.

$$j_{OH^-,lost} = j_{tot} - j_{tot} \cdot WDE + j_{tot}\left(\frac{2}{3}(1 - WDE)\right) \quad (31)$$

$$\frac{j_{OH^-,lost}}{j_{tot}} = \frac{5}{3}(1 - WDE) \quad (32)$$

Using the Faraday's Law of Electrolysis, we can convert Eq. 32 into a molar ratio. Here we aim for the produced CO instead of the total current, which converts j_{tot} into j_{CO}/FE(CO). In the derivation, the number of electrons for CO to OH^- is 2. Finally, the ratio is converted into mass using the molar masses of KOH and CO, noting that 1 mol of OH^- is equal to 1 mol of KOH.

$$\frac{j_{OH^-,lost}}{(\frac{j_{CO}}{FE_{CO}})} = \frac{\left(\frac{\dot{n}_{OH^-,lost} z_{OH^-} F}{Area}\right)}{\left(\frac{\dot{n}_{CO} z_{CO} F}{Area}\right) \cdot \frac{1}{FE_{CO}}} = \frac{5}{3}(1 - WDE) \quad (33)$$

$$\frac{\dot{n}_{OH^-,lost}}{\dot{n}_{CO}} = \frac{n_{e^-,CO}}{n_{e^-,OH}}(1 - WDE) = \frac{2}{FE_{CO}}\frac{5}{3}(1 - WDE) \quad (34)$$

$$\frac{\dot{n}_{OH^-,lost}}{\dot{n}_{CO}} = \frac{\dot{n}_{KOH,lost}}{\dot{n}_{CO}} = \frac{\frac{\dot{m}_{KOH,lost}}{M_{KOH}}}{\frac{\dot{m}_{CO}}{M_{CO}}} = \frac{2}{FE_{CO}}\frac{5}{3}(1 - WDE) \qquad (35)$$

$$\frac{\dot{m}_{KOH,lost}}{\dot{m}_{CO}} = \frac{10}{3}\frac{(1-WDE)}{FE(CO)} \cdot \frac{M_{KOH}}{M_{CO}} \qquad (36)$$

This equation is used to produce Supplementary Fig. 7. Thus, from Eq. 36 when WDE = 90% and FE(CO) = 60%, then the ratio of lost KOH to produced CO is 1.1.

Supplementary Note 5 - CO2 electrolysis performance evaluation

The Faradaic efficiencies for the gas products were calculated using Eq. 37:

$$FE_{i,gas} = \frac{z \cdot P \cdot Q_{gas} \cdot c_i \cdot F}{R \cdot T \cdot I} \qquad (37)$$

Where P is the pressure (101325 Pa), Q_{gas} is the volumetric flow rate of the gases and c_i the concentration of product i measured by the GC. F corresponding to the Faraday constant (96485 $C \cdot mol^{-1}$), R is the gas constant (8.3145 $m^3 \cdot Pa \cdot K^{-1} \cdot$mol-1, T the temperature, z the number of charges transferred in the electrochemical reaction, and I the total current.

The volumetric flow rate of the gases, Q_{gas}, is measured by a mass flow meter calibrated for CO_2. To convert the measured CO_2-calibrated flowrate from to flowrate (Q_{gas}) we use the following equations:

$$Q_{gas} = \frac{Q_{MFM}}{C_{CO_2}} C_{mix} \qquad (38)$$

$$C_{mix} = \frac{1}{\sum_i \frac{c_i}{C_i}} \qquad (39)$$

Q_{MFM} is obtained from the last 30 seconds flowrates registered by the MFM before a GC injection. C_{mix} is the gas mixture conversion factor obtained from the volume fraction of the gases (c_i) and their gas conversion factor (C_i), being 0.74 for CO_2, 1.00 for CO, 1.01 for H_2 and 0.79 for H_2O vapour. It is assumed 100% relative humidity.

Supplementary Data

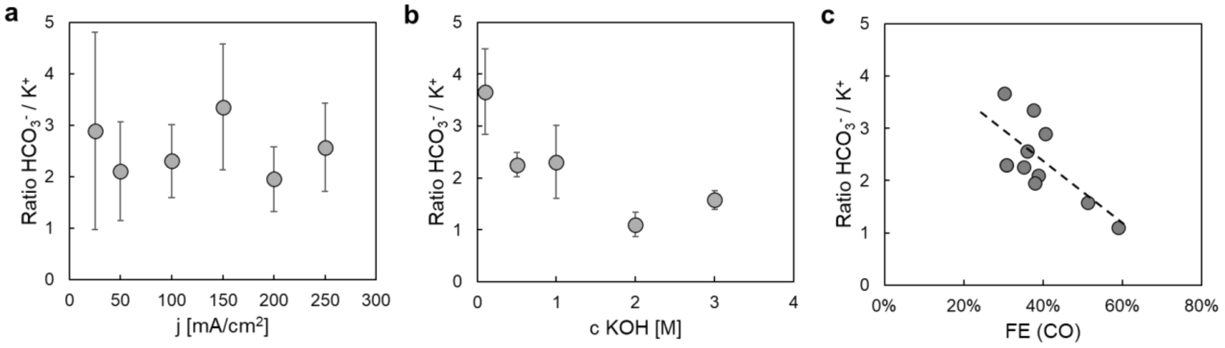

Fig. 1. Co-ion crossover ratio**.** Ratio of HCO_3^- vs K^+ as a function of **a.** current density (average of 2) and **b.** KOH concentration. **c.** relationship between Ratio of HCO_3^- / K^+ and the FE(CO).

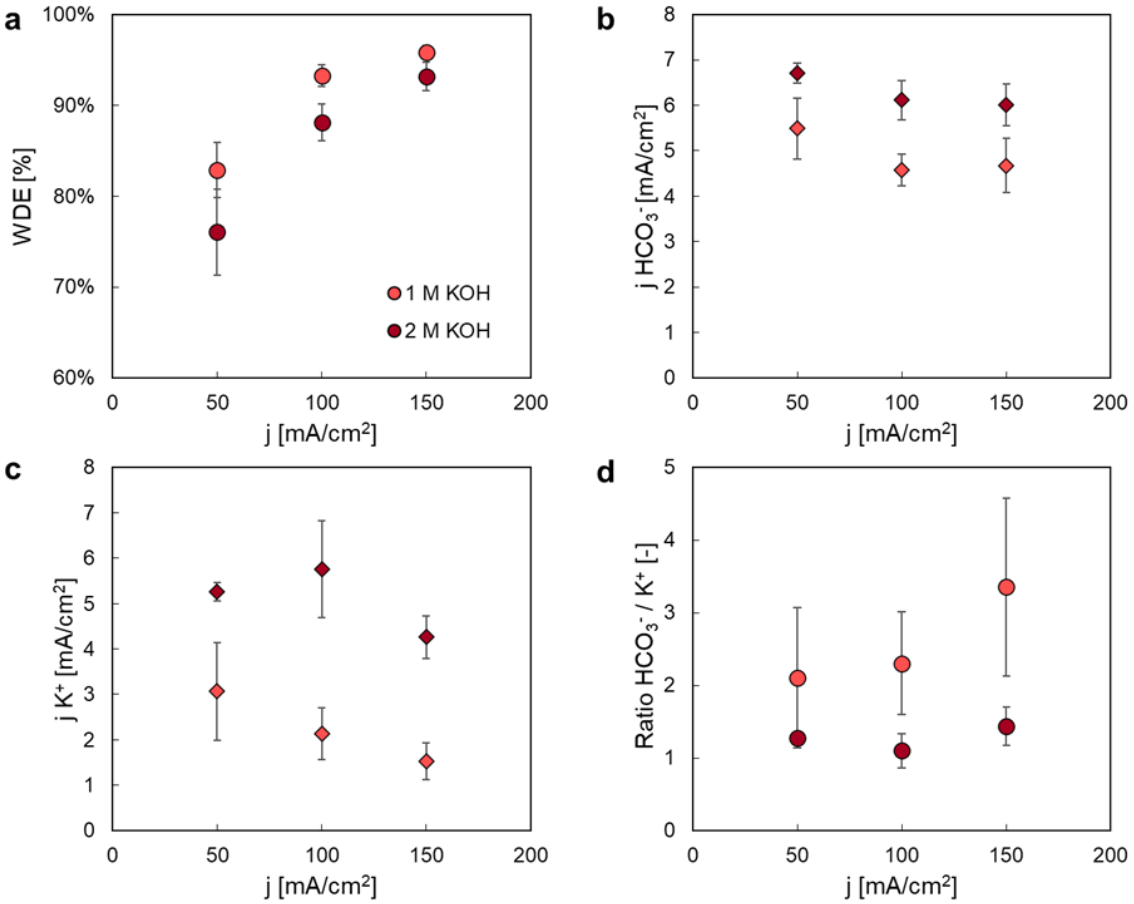

Fig. 2. Comparing the effect of anolyte concentration (1 M KOH and 2 M KOH) vs current density (50, 100 and 200 mA·cm^{-2}). **a.** WDE, **b.** $j_{HCO_3^-}$ **c.** j_{K^+}. **d.** ratio of HCO_3^- / K^+.

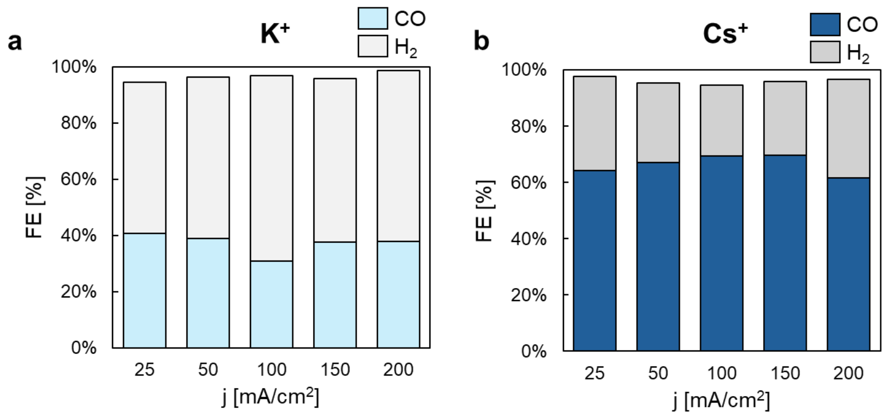

Fig. 3. Faradaic Efficiency of CO and H_2 for 1M **a.** KOH and **b.** CsOH anolytes at different current densities 25-200 mA·cm^{-2}.

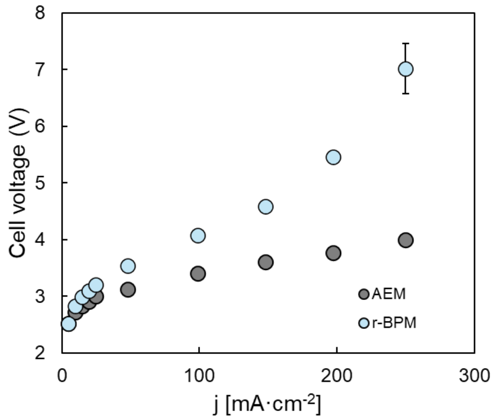

Fig. 4. Cell voltage of a an AEMEA and a r-BPMEA CO_2 electrolyzer as a function of current density 25-250 mA·cm^{-2}. AEMEA with a PiperIon 40µm, a Ag 100 nm GDE (Sigracet 39BB) and an IrO_2 anode (HPNow) using a 1 M $KHCO_3$ anolyte.

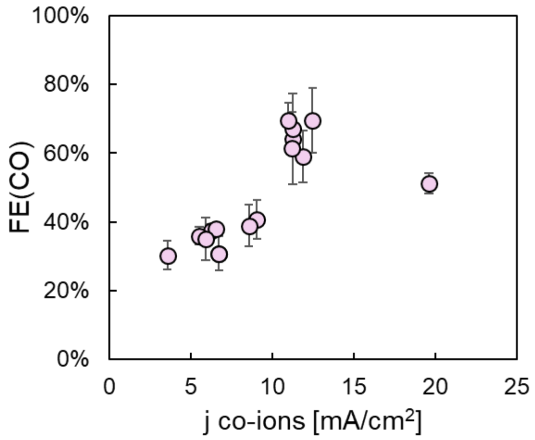

Fig. 5. Trade-off between co-ion transport and CO2R selectivity towards CO in r-BPMEA CO_2 electrolyzer. All data presented in Fig. 3 and Fig. 4 in the manuscript is included.

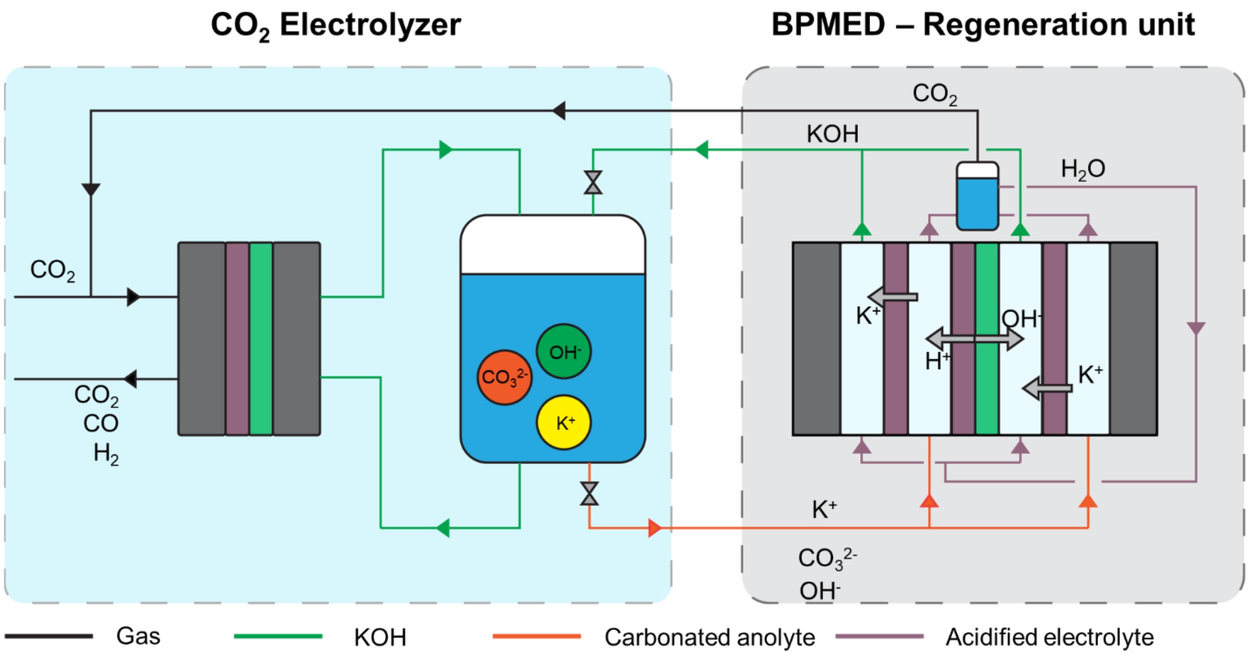

Fig. 6. BPMED anolyte regeneration unit integrated for a BPM CO_2 electrolyzer.

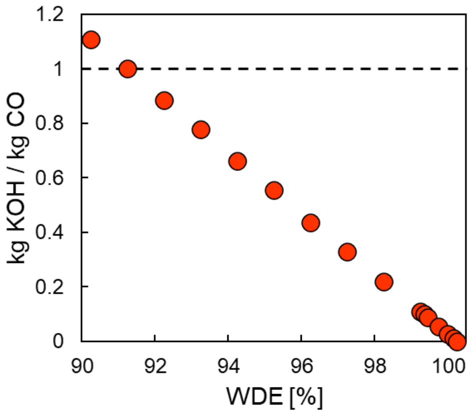

Fig. 7. WDE dependency on the amount of KOH required per kg of CO produced. It is assumed a FE(CO) = 60%.

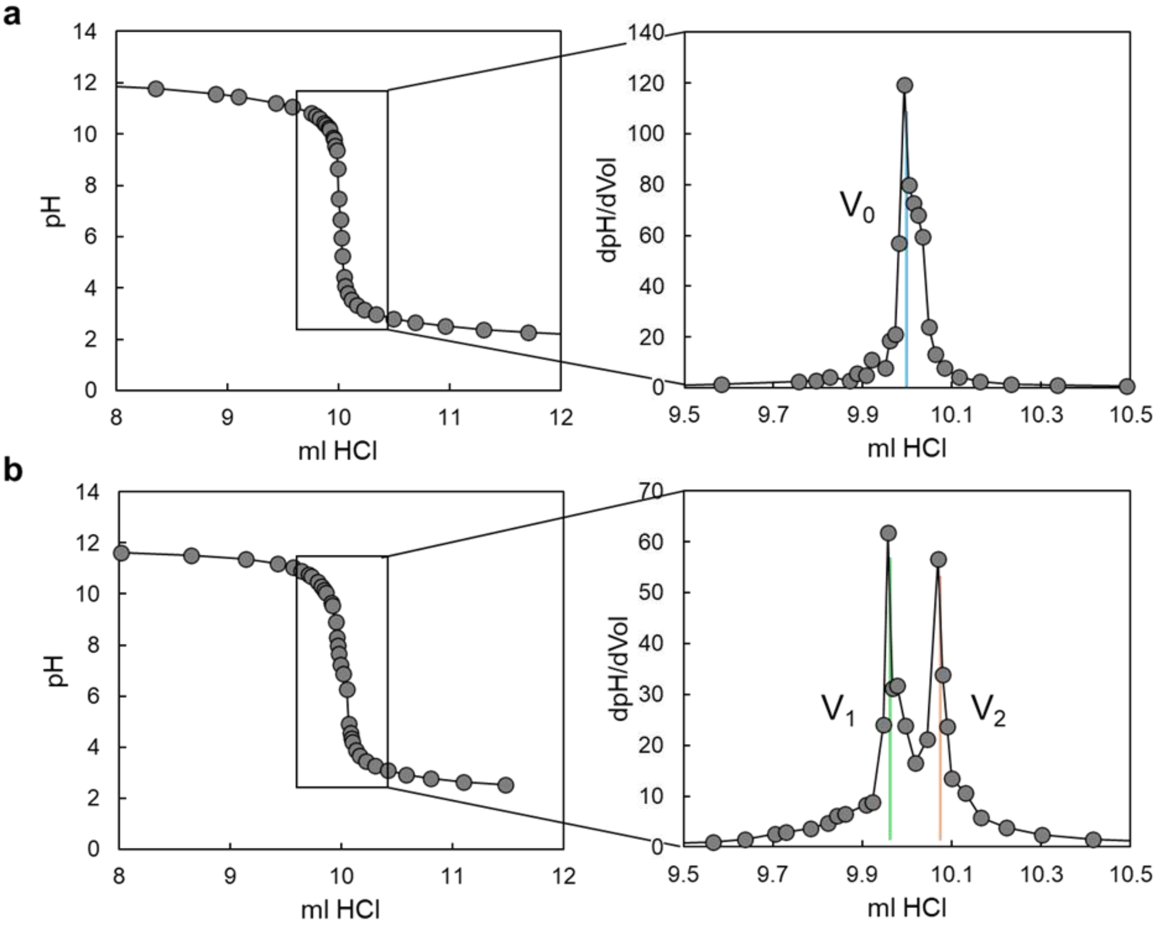

Fig. 8. Example of the titration of an anolyte sample **a.** before and **b.** after CO_2 electrolysis. Equivalence points obtained for determining the concentration of OH^- and CO_3^{2-}.

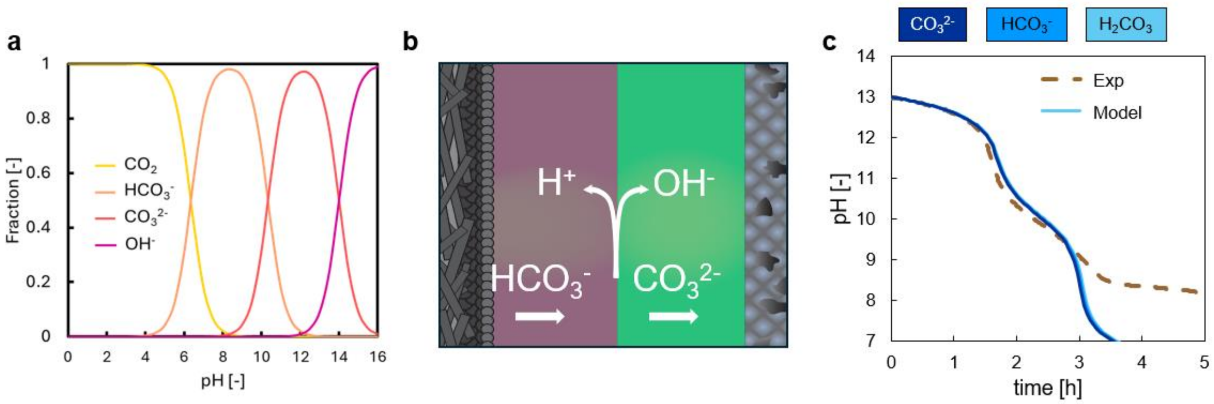

Fig. 9. Which carbon specie is crossing through the BPM? a. Carbon species distribution as a function of pH. **b.** Assuming HCO_3^- as carbon specie crossing the CEL and converted to CO_3^{2-} in the AEL. **c.** Modelling verification with a experiment (20 ml 0.1 M KOH at 100 mA·cm^{-2}) in which it is determined that the carbon specie considered in the model has not any effect on the prediction of pH shift.

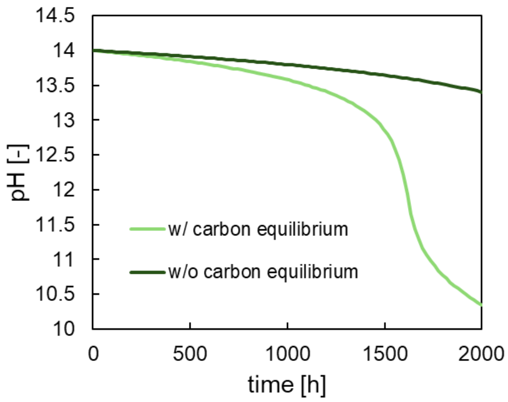

Fig. 10. Modelling output differences when carbonate crossover and buffering effects are considered in the anolyte tank. The model represents a r-BPMEA CO_2 electrolyzer operating with a WDE = 99% at 200 mA·cm^{-2}, and with a 1 L of 1 M KOH anolyte.

References

1. Allen J. Bard, Larry R. Faulkner & Henry S. White. *Electrochemical Methods - Fundamentals and Applications*. (Wiley, 2022).

2. Li, M. *et al.* Local ionic transport enables selective PGM-free bipolar membrane electrode assembly. *Nature Communications* **15**, (2024).

3. Yue, P. *et al.* Microenvironment Regulation Strategies Facilitating High-Efficiency CO2 Electrolysis in a Zero-Gap Bipolar Membrane Electrolyzer. *ACS Appl Mater Interfaces* **15**, 53429–53435 (2023).

4. Schulz, K. G., Riebesell, U., Rost, B., Thoms, S. & Zeebe, R. E. Determination of the rate constants for the carbon dioxide to bicarbonate inter-conversion in pH-buffered seawater systems. *Mar Chem* **100**, 53–65 (2006).
